# Subcostal single-port robot-assisted resection of a posterior mediastinal tumor in an infant

**DOI:** 10.1007/s11748-026-02356-x

**Published:** 2026-07-08

**Authors:** Teruhisa Kawaguchi, Fumiaki Watanabe, Shinji Kaneda, Daisuke Ito, Kohei Matsushita, Koji Kawaguchi

**Affiliations:** 1 Department of Thoracic Surgery, Mie University, 2-174 Edobashi, Tsu, Mie 514-8507 Japan; 2 Department of Gastrointestinal and Pediatric Surgery, Mie University, 2-174 Edobashi, Tsu, Mie 514-8507 Japan

**Keywords:** Single-port robotic surgery, Pediatric thoracic surgery, Subcostal approach

## Abstract

Robot-assisted thoracic surgery is increasingly used in pediatric patients; however, its application in infants weighing less than 8 kg remains limited. We report a case of posterior mediastinal tumor resection in a 6-month-old female infant weighing 5.46 kg. A subcostal approach using the da Vinci Single Port system was employed to minimize instrument collision within the small thoracic cavity. A 3.5-cm subcostal incision was created, and the access port was secured without intercostal rib manipulation. The operative field was maintained using CO_2_ insufflation at 3 mmHg. A 7.0-cm cystic tumor was successfully resected. The total operative time was 132 min. This intercostal-sparing technique may reduce intercostal nerve injury and subsequent long-term musculoskeletal complications. Subcostal single-port robotic surgery appears to be a safe and feasible minimally invasive option for infants weighing less than 8 kg.

**Supplementary Information:**

The online version contains supplementary material available at https://doi.org/10.1007/s11748-026-02356-x.

## Introduction

The use of RATS in pediatric surgery is increasing. But there are no reports of its application in infants weighing 8 kg or less. In addition, the intercostal approach in children can be associated with long-term musculoskeletal complications [1]. We recently reported a subcostal approach using the da Vinci SP system (da Vinci Surgical Systems, Inc., Sunnyvale, CA, USA) for a giant mediastinal tumor in an 8-year-old child [2]. Based on this experience, we thought that the subcostal approach would allow effective utilization of the thoracic cavity even in small infants.

## Patient

**Fig. 1:**
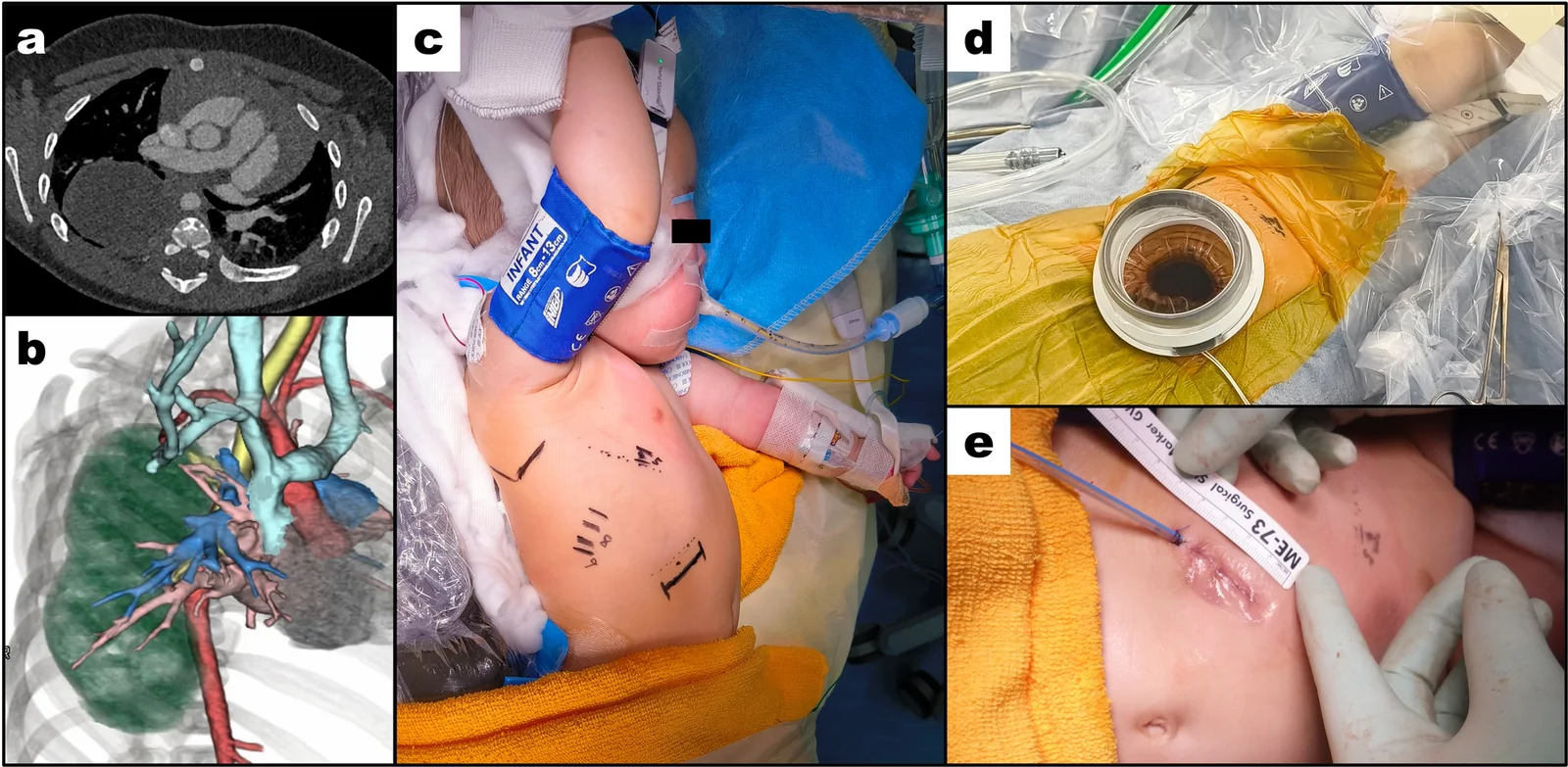
**a**, **b** A computed tomography scan revealed cystic lesion in the posterior mediastinum like snowman configuration. We created a 3D model of the blood vessels and bronchi. No obvious anomalies were found. **c** The patient was placed in the left lateral decubitus position. **d** We secured a small access port. **e** A 10-Fr. drain was left in place after the procedure

A 6-month-old female infant (weight, 5.46 kg; height, 59.3 cm) was referred to our department with a right-sided posterior mediastinal mass (Fig. 1a, b). Prior to surgery, our institutional Medical Safety Committee reviewed the high-risk procedure, and with the hospital director’s approval (approval No. 70). Written informed consent was obtained from the parents after a full explanation of the procedure.

### Operation

The surgery was performed under general anesthesia and an epidural block. One-lung ventilation was achieved using a bronchial blocker and a 4.5-mm internal diameter uncuffed endotracheal tube. The patient was placed in the left lateral decubitus position (Fig. 1c). A 3.5 cm transverse skin incision was made along the right 8th rib subcostal margin (Video 1). The abdominal muscles were incised, and dissection continued toward the inner surface of the thoracic cage. Establishing the access route was more difficult than in adults because of the high elasticity of the peritoneum and the soft, flexible nature of the infant’s ribs.

A dedicated small-sized access port kit for the da Vinci SP was placed after the diaphragm had been sutured to the port site (Fig. 1d). CO_2_ insufflation was maintained at a pressure of 3 mmHg. The first and second arms were equipped with Maryland bipolar forceps, and the third arm was equipped with a Cadiere forceps. Intraoperatively, a 7.0 cm fluid-filled cystic mass covered with a thick capsule was identified on the right side of the vertebral body, completely separate from the lung. The pleura over the tumor was incised, and the mass was carefully dissected from the esophagus and descending aorta. The right vagus nerve was clipped and divided due to its involvement with the tumor. The tumor was resected en bloc with its capsule intact. The diaphragm was sutured, and a 10-Fr drain was placed (Fig. 1e).

The total operative time was 132 min. The time from skin incision to port placement was 41 min, and console time was 55 min. Blood loss was minimal. The patient had bloody stool on day 1, which resolved by day 2. Consequently, the parents declined further evaluation. *The epidural block continued until day 2*. The chest drain was removed on day 3, and the patient was discharged on day 4. Histopathology confirmed a mature teratoma measuring 7.0 cm in diameter. No complications were observed at the 12-day follow-up visit.

## Discussion

The feasibility of pediatric RATS is being increasingly recognized, but a weight threshold of 8 kg is often considered a limitation for traditional intercostal approaches [3–5]. Our experience suggests that the subcostal approach using the da Vinci SP system can overcome this barrier by providing a unique surgical corridor even in a 5.5-kg infant. Several technical points are critical for performing this procedure safely in small infants.

First, access port placement requires extra care. Unlike adults and our 8 year-old case, infants have high peritoneal elasticity and very soft, flexible ribs. Additionally, because the liver is relatively large in infants, the subcostal space is narrow. As a technical tip, we recommend placing the skin incision slightly more cranially than in adults to avoid the liver and secure a direct route to the diaphragm.

Second, space creation and instrument management are key. In a small thoracic cavity, instrument clashing is a major concern. However, once the SP port is established, its coaxial design eliminates external clashing. To maintain the working space, we used a CO_2_ insufflation pressure of 3 mmHg after consulting with the anesthesiologist. We found that 3 mmHg combined with selective lung ventilation provided a stable and sufficient surgical field. If one-lung ventilation was difficult, we planned to proceed with insufflation alone.

In conclusion, the subcostal single-port approach maximizes the advantages of the da Vinci SP system’s 3D vision and multi-jointed arms, allowing for safe dissection even near vital structures. Further studies are needed to evaluate whether this method is minimally invasive, including refining the port placement technique and assessing long-term outcomes.

## Supplementary Information

Below is the link to the electronic supplementary material.

Video 1: Video of da Vinci SP surgery for the posterior mediastinal tumor
